# Antiplatelet Prescription in Atrial Fibrillation: Association with a Low Rate of Anticoagulation

**DOI:** 10.1055/s-0038-1660506

**Published:** 2018-06-07

**Authors:** Romain Chopard, Samuel Z. Goldhaber, Neelima Karipineni, Howard S. Goldberg, Gregory Piazza

**Affiliations:** 1 Cardiovascular Division, Department of Medicine, Brigham and Women's Hospital, Harvard Medical School, Boston, Massachusetts, United States; 2 Division of General Internal Medicine, Brigham and Women's Hospital, Harvard Medical School, Boston, Massachusetts, United States

Anticoagulants reduce the risk of thromboembolism and death in patients with atrial fibrillation (AF). 1 2 Evidence-based clinical practice guidelines recommend anticoagulation based on the CHA _2_ DS _2_ -VASc score to decrease the risk of stroke in patients with AF. 3 Conversely, randomized trials demonstrate the inadequacy of antiplatelet agents compared with anticoagulants for stroke prevention in at-risk AF patients. 4 5 We have reported in our AF outpatient cohort study that anticoagulants were prescribed in half of the patients with an indication for anticoagulation. 6 We now analyze this database to determine clinical factors associated with non-prescription of anticoagulation in AF.

We reviewed 5,062 consecutive records of patients who had the diagnosis of AF entered into the electronic health record within our outpatient clinic network between March 2013 and March 2014. We utilized the 2011 American College of Cardiology/American Heart Association/Heart Rhythm Society guidelines, which recommended anticoagulation in AF patients with a CHA _2_ DS _2_ -VASc score ≥2, 3 to narrow our final cohort to 3,677 patients. The aim of the study was to identify factors independently associated with nonprescription of anticoagulation for stroke prevention in AF patients with CHA _2_ DS _2_ -VASc score ≥2. Anticoagulation was defined as the prescription of any United States Food and Drug Administration–approved anticoagulant therapy for stroke prevention in patients with AF. Vascular disease included coronary artery disease (CAD) and peripheral arterial disease (PAD). Bleeding risk was estimated with the modified HAS-BLED score, excluding fluctuation of the International Normalized Ratio (INR) because the computer program could not search for labile INR. 7 To investigate associations with the nonprescription of anticoagulation, we undertook a multivariate analysis by constructing hierarchical modified Poisson's regression models with robust error variance, adjusted for patient- and practice-level characteristics with a *p* -value of <0.10 by univariate analysis. 8 Covariates entered into the model were age, sex, body mass index (BMI), race, ethnicity, hypertension, diabetes mellitus, heart failure (HF), CAD, PAD, chronic kidney disease, prior stroke, liver disease, prior major bleeding, alcoholism, and antiplatelet therapy. A relative risk greater than 1 corresponds to an increased probability of anticoagulation nonprescription. We identified factors related to prescription of antiplatelet therapy by using the same method. These models included site as a random effect to account for patient clustering within sites. Statistical tests were two sided and considered significant if they yielded a *p* -value of <0.05.

In total, 1,945 (52.9%) patients were prescribed anticoagulation and 1,732 (47.1%) were not. Table 1 summarizes the baseline characteristics and antiplatelet regimen of the study population. Patients who were prescribed anticoagulation were older, had higher BMI, and presented with higher rates of hypertension, HF, and prior stroke compared with those who did not receive anticoagulation by univariate analysis. These differences were reflected by higher CHA _2_ DS _2_ -VASc scores in the anticoagulant patient group (median [IQR]: 3 [3–5] vs. 3 [2–4]; *p* < 0.001). Antiplatelet therapy was prescribed for 40.7% of patients not receiving anticoagulation and for 25.9% of those receiving anticoagulation ( *p* < 0.001). Concomitant antiplatelet and anticoagulant therapies were more frequently prescribed in patients with vascular disease (48.5 vs. 20.9%; *p* < 0.001). The proportion of patients receiving anticoagulation (with and without an antiplatelet agent) increased with CHA _2_ DS _2_ -VASc score ( *p* < 0.001) and with HAS-BLED score ( *p* < 0.001).

**Table 1:** Baseline characteristics and antiplatelet regimen of atrial fibrillation patients at risk of stroke as categorized by the 2011 American College of Cardiology/American Heart Association/Heart Rhythm Society (CHA _2_ DS _2_ -VASc score ≥2) guidelines, stratified by prescription and nonprescription of anticoagulants

Characteristics	CHA _2_ DS _2_ -VASc score ≥2 ( *N* = 3,677)		*p* -Value
	Anticoagulation		
	Yes ( *N* = 1,945)	No ( *N* = 1,732)	
Mean age ± SD, y	75.5 ± 10.0	74.0 ± 10.7	0.11
Male (%)	1,039 (53.4)	885 (51.1)	0.16
Mean BMI ± SD, kg/m ^2^	29.3 ± 6.5	28.2 ± 6.1	<0.001
Race (%)			
White	1,586 (81.5)	1,442 (83.3)	0.17
Black	125 (6.4)	81 (4.7)	0.02
Other	117 (6.0)	91 (5.2)	0.35
Clinical history (%)			
Hypertension	1,459 (75.0)	1,205 (69.6)	<0.001
Diabetes mellitus	459 (23.6)	367 (21.2)	0.08
Heart failure	463 (23.8)	309 (17.8)	<0.001
CAD	574 (29.5)	514 (29.7)	0.91
Valvular heart disease	361 (18.6)	293 (16.9)	0.19
PAD	127 (6.5)	98 (5.7)	0.30
CKD	189 (9.7)	194 (11.2)	0.14
Prior stroke	293 (15.1)	149 (8.6)	<0.001
Liver disease	30 (1.5)	31 (1.8)	0.60
Prior major bleeding	104 (5.3)	91 (5.2)	0.94
Alcoholism	31 (1.6)	37 (2.1)	0.26
Median CHA _2_ DS _2_ -VASc score (Q1–Q3), points	3 (3–5)	3 (2–4)	<0.001
Median HAS-BLED score (Q1–Q3), points	2 (2–3)	2 (2–3)	0.21
Any antiplatelet (%)	503 (25.9)	705 (40.7)	<0.001
Dual-antiplatelet therapy (%)	40 (2.3)	<0.001	<0.001

Abbreviations: BMI, body mass index; CAD, coronary artery disease; CKD, chronic kidney disease; PAD, peripheral artery disease; SD, standard deviation.

The rate of prescription of antiplatelet therapy alone did not vary significantly according to the CHA _2_ DS _2_ -VASc score, between 17.2% for the CHA _2_ DS _2_ -VASc score of 2 and 22.1% for the CHA _2_ DS _2_ -VASc score of 5 ( *p* = 0.72). In contrast, the rate of antiplatelet prescription increased with the HAS-BLED score from 13.9% for HAS-BLED score of 1 to 54.1% for HAS-BLED score of 5 to 7 ( *p* < 0.001).

**Fig. 1:**
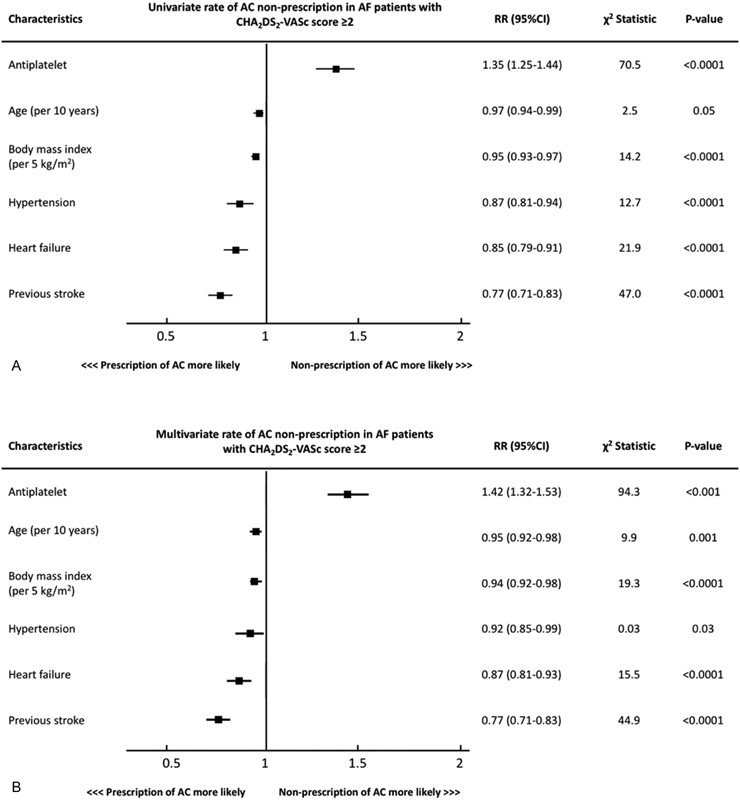
Multivariate analysis of factors associated with anticoagulant nonprescription versus prescription in patients with atrial fibrillation at risk of stroke (CHA _2_ DS _2_ -VASc score ≥2). AC, anticoagulant; AF, atrial fibrillation; CI, confidence interval; RR, relative risk.

After multivariate adjustment, the only factor independently associated with non-prescription of anticoagulation was antiplatelet therapy (relative risk [RR] = 1.42; 95% confidence interval [CI] = 1.32–1.53). Increased age (RR = 0.95; 95% CI = 0.92–0.98), increased BMI (RR = 0.94; 95% CI = 0.92–0.98), hypertension (RR = 0.92; 95% CI = 0.85–0.99), HF (RR = 0.87; 95% CI = 0.81–0.93), and previous stroke (RR = 0.77; 95% CI = 0.71–0.83) were factors associated with increased frequency of anticoagulation prescription ( Fig. 1 ). Male sex (RR = 1.05; 95% CI = 1.009–1.11), hypertension (RR = 1.08; 95% CI = 1.03–1.14), and CAD (RR = 1.52; 95% CI = 1.42–1.64) were associated with prescription of antiplatelet therapy.

Overall, we observed an inverse relationship between antiplatelet prescription and anticoagulant nonprescription. Other investigators have reported alcohol abuse, cancer, and falls as factors related to nonprescription of anticoagulation in addition to antiplatelet prescription. 9

In our analysis, the rate of antiplatelet prescription alone was constant, regardless of stroke risk as defined by the CHA _2_ DS _2_ -VASc score. This suggests that antiplatelet therapy might have been prescribed for an indication other than stroke prevention, such as prevention of cardiovascular events, especially in patients with known vascular disease. Indeed, we identified risk factors for atherosclerosis (i.e., male gender and hypertension), along with CAD itself, as factors associated with prescription of antiplatelet therapy. An analysis of the PINNACLE registry found, besides hypertension and CAD, that dyslipidemia and PAD were factors associated with prescription of aspirin rather than anticoagulation. 10 Furthermore, the high rate of concomitant prescription of anticoagulant and antiplatelet agents in patients with vascular disease may reflect providers' concerns regarding the high risk of future vascular complications. We observed an increase in antiplatelet prescription alone in patients with a high HAS-BLED score, potentially because of concerns about causing bleeding with anticoagulant therapy. Other possible reasons for antiplatelet therapy prescription may include primary prevention in patients at high risk of cardiovascular disease, prevention of colon cancer, 11 or patient's refusal of anticoagulation.

Our analysis has limitations. The precise reason that prompted the providers to prescribe antiplatelet therapy instead of guidelines-recommended anticoagulation was not recorded. The frailty status and life expectancy of the patients were not recorded. We do not have data regarding potential difficulties with warfarin management, which may have led to its discontinuation. Finally, we were unable to determine whether antiplatelet therapy was prescribed specifically for stroke prevention in AF or for some other reason.

In conclusion, prescription of antiplatelet therapy was the only factor we identified that was associated with omission of anticoagulants in this study of AF outpatients at risk of stroke. Prevention of vascular events or concern for bleeding complications could be motivating factors for antiplatelet prescription rather than anticoagulation prescription. Factors compelling clinicians to prescribe antiplatelet therapy warrant consideration when assessing rates of anticoagulation in patients with AF.
